# Supplementary material for: Heat adaptation of phage T7 under an extended genetic code
Source: Virus Evol. 2021 Dec 1;7(2):veab100. doi: 10.1093/ve/veab100 (PMC8923235; doi:10.1093/ve/veab100)
Supplement: veab100_Supp [file veab100_supp.zip › Sequencing_Analysis.zip!/Sequencing_Analysis/S3/evidence/JC_SIDE_1_49.html]

BRESEQ :: Evidence


| New junction evidence | | | | | | | | | | | |
| --- | --- | --- | --- | --- | --- | --- | --- | --- | --- | --- | --- |
|  | | seq id | position | reads (cov) | reads (cov) | score | skew | freq | annotation | gene | product |
| \* | ? | NC\_001604.1 | 8888 = | 2048 (1.190) | 3 (0.000) | 3/592 | 47.8 | 0.15% | intergenic (–/–) | *–/–* | –/– |
|  | ? | NC\_001604.1 | 8897 = | 2013 (1.190) |  |  |  |  | intergenic (–/–) | *–/–* | –/– |
| Rejected: Coverage evenness skew score above cutoff. | | | | | | | | | | | |
| Rejected: Frequency below/above cutoff threshold. | | | | | | | | | | | |

Only 100 of 2078 total aligned reads displayed.  

`TCGTTCAATGACCGTTGAGCAGTTCAAGGGATACTGCTTCGGTAACATCTTAAAGTACAGACTACGTGCTGGTAAGAAGTCAGAGTTAGCGTACTTAGAGAAAGACCTAGCGAAAGCAGACTTCTATAAAGAACTCTTTGAGAAACATAAGGATAAATGTTATGCATAACTTCAAGTCAACCCCACCTGCCGACAGCCTATCTGATGACTTCACATCTTGCTCAGAGTGGTGCCGAAAGATGTGGGAAGAGACATTCGACGATGCGTACATCAAGCTGTATGAACTTTGGAAATCGAGAGGTCAATGACTATGTCAAACGTAAATACAGGTTCACTTAGTGTGGACAATAAGAAGTTTTGGGCTACCGTAGAGTCCTCGGAGCATTCCTTCGAGGTTCCAATCTACGCTGAGACCCTAGACGAAGCTCTGGAGTTAGCCGAATGGCAATACGTTCCGGCTGGCTTTGAGGTTACTCGTGTGCGTCCTTGTGTAGCACCGAAGTAATACGACTCACTATTAGGGAAGACTCCCTCTGAGAAACCAAACGAAACCTAAAGGAGATTAACATTATGGCTAAGAAGATTTTCACCTCTGCGCTGG  >  NC_001604.1/8588‑9188`  

                                                                                                                                                                                                                                                                                                            `|`                                                                                                                                                                                                                                                                                                              
       `aTGACCGTTGAGCAGTTCAAGGGATACTGCTTCGGTAACATCTTAAAGTACAGCCTACGTGCCGGTAAGAAGTCAGAGTTAGCGTACTTAGAGAAAGACCTAGCGAAAGCAGACTTCTATAAAGAACTCTTTGAGAAACATAAGGATAAATGTTATGCATCACTTCAAGTCAACCACACCTGCCGACAGCCTATCTGATGACTTCACATCTTGCTCAGAGTGGTGCCGAAAGATGTGGGAAGAGACATTCGACGATGCTTACATCAAGTTGTATGAACTTTGGACATCGAGAGTTCAATGa                                                                                                                                                                                                                                                                                                       >  1:150757/1‑301 (MQ=255)`  
       `aTGACCGTTGAGCAGTTCAAGGGATACTGCTTCGGTAACATCTTAAAGTACAGACTACGTGCTGGTAAGAAGTCAGAGTTAGCGTACTTAGAGAAAGACCTAGCGAAAGCAGACTTCTATAAAGAACTCTTTGAGAAACATAAGGATAAATGTTATGCATAACTTCAAGTCAACCCCACCTGCCGACAGCCTATCTGATGACTTCACATCTTGCTCAGATTGGTGCCGAAAGATGTGGGAAGAGACATTCGACGAGGCGTACATCAAGCTGTATGAACTTTGGAAATAGAGAGGTCAATGa                                                                                                                                                                                                                                                                                                       >  1:344463/1‑301 (MQ=255)`  
        `tGACCGTTGAGCAGGTCAAGTGATACTGCTTCGGTAACATCTTAAAGTACAGACTACGTGCTGGTAAGAAGTCAGAGTTAGCGTACTTAGAGAAAGACCTAGCGAAAGCAGACTTCTATAAAGAACTCTTTGAGAAACATAAGGATAAAGGGTATGCATAACGTCAAGTCAACCCCACCAGCCGACAGCCTATCTGATGACTTCACATCTTGCTCAGAGTGGGGCCGAAAGATGTGGGAAGAGACATTCGACGATGCGTACATCAAGCTGTATGAACTTTGGAAATCGAGAGGTCAATGAc                                                                                                                                                                                                                                                                                                      <  1:341593/301‑1 (MQ=255)`  
        `tGACCGTTCAGCAGTTCAAGGGATACTGCTTCGGTAACATCTTAAAGTACAGACTACGTTCTGGTAAGAAGTCAGAGTTAGCGTACTTAGAGAAAGACCTAGCGAAAGCAGACTTCTATAAAGAACTCTTTGAGAAACATAAGGATAAATGTTATGCATAACTTCAAGTCAACCCCACCTGCCGACAGCCTATCTGATGACTTCACATCTTGCTCAGAGTGGTGCCGAAAGATGTGGGAAGAGACATTCGACGATGCGTACATCAAGCTGTATGAACTTTGGAAATCGAGAGGTCAATGAc                                                                                                                                                                                                                                                                                                      <  1:72693/301‑1 (MQ=255)`  
        `agACCGTTGAGCAGTTCAAGGGATACTGCTTCGGTAACATCTTAAAGTACAGACTACGTGCTGGTAAGAAGTCAGAGTTAGCGTACTTAGAGAAAGACCTAGCGAAAGCAGACTTCTATAAAGAACTCTTTGAGAAACATAAGGATAAATGTTATGCATAACTTCAAGTCAACCCCACCTGCCGACAGCCTATCTGATGACTTCACATCTTGCTCAGAGTGGTGCCGAAAGATGTGGGAAGAGACATTCGACGATGCGTACATCAAGCTGTATGAACTTTGGAAATCGAGAGGTCAATGAc                                                                                                                                                                                                                                                                                                      <  1:79604/300‑1 (MQ=255)`  
           `ccGTTGAGCAGTTCAAGGGATACTGCTTCGGTAACATCTTAAAGTACAGACTACGTGCTGGTAAGAAGTCAGAGTTAGCGTACTTAGAGAAAGACCTAGCGAAAGCAGACTTCTATAAAGAACTCTTTGAGAAACATAAGGATAAATGTTATGCATAACTTCAAGTCAACCCCACCTGCCGACAGCCTATCTGATGACTTCACATCTTGCTCAGAGTGGTGCCGAAAGATGTGGGAAGAGACATTCGACGATGCGTACATCAAGCTGTATGAACTTTGGAAATCGAGAGGTCAATGAcctg                                                                                                                                                                                                                                                                                                   >  1:291944/1‑298 (MQ=255)`  
                `gAGCAGTTCAAGGGATACTGCTTCGGTAACATCTTAAAGTACAGACTACGTGCTGTTACGCAGTCAGAGTTATCGTACTTAGAGACAGCCCTATCGAAAGCAGACTTCTATAAAGAACTCTTTGAGAACCATAAGGATACATGTTATGCATAACTTCAAGTCAACCCCACCTGCCGACAGCCTATCTGATGACTTCACATCTTGCTCAGAGTGGTGCCGAACGATGTGGGAAGAGACATTCGACGATGCGTACATCAAGCTGTATGAACTTTGGAAATCGAGAGGTCAATAAcctgtctct                                                                                                                                                                                                                                                                                              >  1:75588/1‑293 (MQ=255)`  
                   `cAGTTCAAGGGATACTGCTTCGGTAACATCTTAAAGTACAGACTACGTGCTGGTAAGAAGTCAGAGTTAGCGTACTTAGAGAAAGACCTAGCGAAAGCAGACTTCTATAAAGAACTCTTTGAGAAACATAAGGATAAATGTTATGCATAACTTCAAGTCAACCACACCTGCCGACAGCCTATCTGATGACTTCACATCTTGCTCAGAGTGGTGCCGAAAGATGTGGGAAGAGACATTCGACGATGCGGATATCAAGCTGTATGAACTTTGGAAATCGAGAGGTCAATGACTATGTCAAACg                                                                                                                                                                                                                                                                                           >  1:89253/1‑301 (MQ=255)`  
                     `gTTCAAGGGATACTGCTTCGGTAACATCTTAAAGTACAGACTACGTGCTGGTAAGAAGTCAGAGTTAGCGTACTTAGAGAAAGACCTAGCGAAAGCAGACTTCTATAAAGAACTCTTTGAGAAACATAAGGATAAATGTTATTCATAACTTCAAGTCAACCCCACCTGCCGACAGCCTATCTGATGACTTCACATCTTGCTCAGAGTGGTGCTGAAAGATGTGGGAAGAGACATTCGACGATGCGTACATCAAGCTGTATGAACTTTGGAAATCGAGAGGTCAATGACTATGTCAAACGTa                                                                                                                                                                                                                                                                                         >  1:120449/1‑301 (MQ=255)`  
                     `gTTCAAGGGATACTGCTTCGGTAACATCTTAAAGTACAGACTACGTGCTGGTAAGAAGTCAGAGTTAGCGTACTTAGAGAAAGACCTAGCGAAAGCAGACTTCTATAAAGAACTCTTTGAGAAACATAAGGATAAATGTTATGCATAACTTCAAGTCAACCCCACCTGCCGACAGCCTATCTGATGACTTCACATCTTGCTCAGAGTGGTGCCGAAAGATGTGGGAAGAGACATTCGACGATGCGTACATCAAGCTGTATGAACTTTGGAAATCGAGAGGTCAATGACTATGTCAAACGTa                                                                                                                                                                                                                                                                                         >  1:302082/1‑301 (MQ=255)`  
                          `atggAAACTGCTTCGGTAACATCTTAAAGTACAGACTACGTGCTGGTAAGAAGTCAGAGTTAGCGTACTTAGAGAAAGACCTAGCGAAAGCAGACTTCTATAAAGAACTCTTTGAGAAACATAAGGATAAATGTTATGCATAACTTCAAGTCAACCCCACCTGCCGACAGCCTATCTGATGACTTCACATCTTGCTCAGAGTGGTGCCGAAAGATGTGGGAAGAGACATTCGACGATGCGTACATCAAGCTGTATGAACTTTGGAAATCGAGAGGTCAATGACTATGTCAAACGTAAATAc                                                                                                                                                                                                                                                                                    <  1:106348/299‑1 (MQ=255)`  
                          `aGGGATACTGCTTCGGTAACATCTTAAAGTACAGACTACGTGCTGGTAAGAAGTCAGAGTTAGCGTACTTAGAGAAAGACCTAGCGAAAGCAGACTTCTATAAAGAACTCTTTGAGAAACATAAGGATAAATGTTATGCATAACTTCAAGTCAACCCCACCTGCCGACAGCCTATCTGATGACTTCACATCTTGCTCAGAGTGGTGCCGAAAGATGTGGGAAGAGACATTCGACGATGCGTACATCAAGCTGTATGAACTTTGGAAATCGAGAGGTCAATGACTATGTCAAACGTAAATAc                                                                                                                                                                                                                                                                                    <  1:245403/301‑1 (MQ=255)`  
                            `ggATACTGCTTCGGTAACATCTTAAAGTACAGACTACGTGCTGGTAAGAAGTCAGAGTTAGCGTACTTAGAGAAAGACCTAGCGAAAGCAGACTTCTATAAAGAACTCTTTGAGAAACATAAGGATAAATGTTATGCATAACTTCAAGTCAACCCCACCTGCCGACAGCCTATCTGATGACTTCACATCTTGCTCAGAGTGGTGCCGAAAGATGTGGGAAGAGACATTCGACGATGCGTACATCAAGCTGTATGAACTTTGGAAATCGAGAGGTCAATGACTATGTCAAACGTAAATACAg                                                                                                                                                                                                                                                                                  >  1:16167/1‑301 (MQ=255)`  
                             `gaaaCTGCTTCGGTAACATCTTAAAGTACAGACTAAGTGCTGGTAAGAAGTCAGAGTTAGCGTACTTAGAGAAAGACCTAGCGAAAGCAGACTTCTATAAAGAACTCTTTGAGAAACATAAGGATAAAGGTTATGCATAACTTCAAGTCAACCCCACCTGCCGACAGCCTATCTGATGACTTCACATCTTGCTCAGAGTGGTGCCGAAAGATGTGGGAAGAGACAGTCGACGATGCGTACATCAAGCTGTATGAACTTTGGAAATCGAGAGGTCAATGACTATGTCAAACGTAAATACAgg                                                                                                                                                                                                                                                                                 <  1:114035/298‑1 (MQ=255)`  
                               `aaCTGCTTCGGTAACATCTTAAAGTACAGACTACGTGCTGGTAAGAAGTCAGAGTTAGCGTACTTAGAGAAAGACCTAGCGAAAGCAGACTTCTATAAAGAACTCTTTGAGAAACATAAGGATAAATGTTATGCATAACTTCAAGTCAACCCCACCTGCCGACAGCCTATCTGATGACTTCACATCTTGCTCAGAGTGGTGCCGAAAGATGTGGGAAGAGACATTCGACGATGCGTACATCAAGCTGTATGAACTTTGGAAATCGAGAGGTCAATGACTATGTCAAACGTAAATACAGGtt                                                                                                                                                                                                                                                                               <  1:231669/300‑1 (MQ=255)`  
                                    `cTTCGGTAACATCTTAAAGTACAGACTACGTGCTGGTAAGAAGTCAGAGTTAGCGTACTTAGAGAAAGACCTAGCGAAAGCAGACTTCTATAAAGAACTCTTTGAGAAACATAAGGATAAATGTTATGCATAACTTCAAGTCAACCCCACCTGCCGACAGCCTATCTGATGACTTCACATCTTGCTCAGAGTGGTGCCGAAAGATGTGGGAAGAGACATTCGACGATGCGTACATCAAGCTGTATGAACTTTGGAAATCGAGAGGTCAATGACTATGTCAAACGTAAATACAGGTTCACtt                                                                                                                                                                                                                                                                          <  1:191528/301‑1 (MQ=255)`  
                                    `cTTCGGTAACATCTTAAAGGACAGAAGACGTGATGGTAAGAAGTCAGAGTTAGCGTACTTAGAGAAAGACCTAGCGAAAGCAGACTTATATAAAGAACTCTTTGAGAAACATAAGGATAAATGTTATGCATAACTTCAAGGCAACCCAACCAGCCGACAATCTAACTGATTACTTCACATCTTGCTCAGAGTGGTGCCAAAAGATGGGGGAAGAGACATTCGACGATGCGTACATCAAGCTGTATGAACTTTGGAAATCGAGAGGTCAACGACTATGTCAAACGTAAATACAGGTTCACtt                                                                                                                                                                                                                                                                          <  1:53720/301‑1 (MQ=255)`  
                                       `cGGTAACATCTTAAAGTACAGACTACGTGCTGGTAAGAAGTCAGAGTTAGCGTACTTAGAGAAAGACCTAGCGAAAGCAGACTTCTATAAAGAACTCTTTGAGAAACATAAGGATAAATGTTATGCATAACTTCAAGTCAACCCCACCTGCCGACAGCCTATCTGATGACTTCACATCTTGCTCAGAGTGGTGCCGAAAGATGTGGGAAGAGACATTCGACGATGCGTACATCAAGCTGTATGAACTTTGGAAATCGAGAGGTCAATGACTATGTCAAACGTAAATAcctgtctcttatac                                                                                                                                                                                                                                                                       >  1:213609/1‑288 (MQ=255)`  
                                        `gggaaCATCTTAAAGTACAGACTACGTGCTGGTAAGAAGTCAGGGTTAGCGTACTTAGCGAAAGACCTAGCGAAAGCAGACTTCTATAAAGAACTCTTTGAGAAACATAACGATAAATGTTATGCATAACTTCAAGTCAACCCCACCTGCCGACAGCCTATCTGATGACTTCACATCTTGCTCAGAGTGGTGCCGAAAGATGTGGGAAGAGACATTCGACGATGCGTACATCAAGCTGTATGAACTTTGGAAATCGAGAGGTCAATGACTATGTCAAACGTAAATACAGGTTCACTTAgtg                                                                                                                                                                                                                                                                      <  1:289379/298‑1 (MQ=255)`  
                                        `ggTAACATCTTAAAGTACAGACTACGTGCTGGTAAGAAGTCAGAGTTAGCGTACTTAGAGAAAGACCTAGCGAAAGCAGACTTCTATAAAGAACTCTTTGAGAAACATAAGGATAAATGTTATGCATAACTTCAAGTCAACCCCACCTGCCGACAGCCTATCTGATGACTTCACATCTTGCTCAGAGTGGTGCCGAAAGATGTGGGAAGAGACATTCGACGATGCGTACATCAAGCTGTATGAACTTTGGAAATCGAGAGGTCAATGACTATGTCAAACGTAAATACAGGTTCACTTAgtg                                                                                                                                                                                                                                                                      <  1:3361/301‑1 (MQ=255)`  
                                        `gacagcATCTTAAAGTACAGACTACGTGCTGGTAAGAAGTCAGAGTTAGCGTACTTAGAGAAAGACCTAGCGAAAGCAGACTTCTATAAAGAACTCTTTGAGAAACATAAGGATAAATGTTATGCATAACTTCAAGTCAACCCCACCTGCCGACAGCCTATCTGATGACTTCACATCTTGCTCAGAGTGGTGCCGAAAGATGTGGGAAGAGACATTCGACGATGCGTACATCAAGCTGTATGAACTTTGGAAATCGAGAGGTCAATGACTATGTCAAACGTAAATACAGGTTCACTTAgtg                                                                                                                                                                                                                                                                      <  1:270977/296‑1 (MQ=255)`  
                                        `aagacagtCTTAAAGTACAGACTACGTGCTGGTAAGAAGTCAGAGTTAGCGTACTTAGAGAAAGACCTAGCGAAAGCAGACTTCTATAAAGAACTCTTTGAGAAACATAAGGATAAATGTTATGCATAACTTCAAGTCAACCCCACCTGCCGACAGCCTATCTGATGACTTCACATCTTGCTCAGAGTGGTGCCGAAAGATGTGGGAAGAGACATTCGACGATGCGTACATCAAGCTGTATGAACTTTGGAAATCGAGAGGTCAATGACTATGTCAAACGTAAATACAGGTTCACTTAgtg                                                                                                                                                                                                                                                                      <  1:285661/294‑1 (MQ=255)`  
                                                    `aggTACAGACTACGTGCTGGTAAGAAGTCAGAGTTAGCGTACTTAGAGAAAGACCTAGCGAAAGCAGACTTCTATAAAGAACTCTTTGAGAAACATAAGGATAAATGTTATGCATAACTTCAAGTCAACCCCACCTGCCGACAGCCTATCTGATGACTTCACATCTTGCTCAGAGTGGTGCCGAAAGATGTGGGAAGAGACATTCGACGATGCGTACATCAAGCTGTATGAACTCTGGAAATCGAGAGGTCAATGACTATGTCAAACGTAAATACAGGTTCACTTAGTGTGGACAATaaga                                                                                                                                                                                                                                                          <  1:252798/299‑1 (MQ=255)`  
                                                     `aGTACAGACTACGTGCTGGTAAGAAGTCAGAGTTAGCGTACTTAGAGAAAGACCTAGCGAAAGCAGACTTCTATAAAGAACTCTTTGAGAAACATAAGGATAAATGTTATGCATAACTTCAAGTCAACCCCACCTGCCGACAGCCTATCTGATGACTTCACATCTTGCTCAGAGTGGTGCCGAAAGATGTGGGAAGAGACATTCGACGATGCGTACATCAAGCTGTATGAACTTTGGAAATCGAGAGGTCAATGACTATGTCAAACGTAAATACAGGTTCCCTTAGTGTGGACAATaagaa                                                                                                                                                                                                                                                         >  1:132952/1‑301 (MQ=255)`  
                                                      `gTACAGACTACGTGCTGGTAAGAAGTCAGAGTTAGCGTACTTAGAGAAAGACCTAGCGAAAGCAGACTTCTATAAAGAACTCTTTGAGAAACATAAGGATAAATGTTATGCATAACTTCAAGTCAACCCCACCTGCCGACAGCCTATCTGATGACTTCACATCTTGCTCAGAGTGGTGCCGAAAGATGTGGGAAGAGACATTCGACGATGCGTACATCAAGCTGTATGAACTTTGGAAATCGAGAGGTCAATGACTATGTCAAACGTAAATACAGGTTCACTTAGTGTGGACAATaagaag                                                                                                                                                                                                                                                        <  1:63850/301‑1 (MQ=255)`  
                                                      `gTACAGACTACGTGCTGGTAAGAAGTCAGAGTTAGCGTACTTAGAGAAAGACCTAGCGAAAGCAGACTTCTATAAAGAACTCTTTGAGAAACATAAGGATAAATGTTATGCATAACTTCAAGTCAACCCCACCTGCCGACAGCCTATCTGATGACTTCACATCTTGCTCAGAGTGGTGCCGAAAGATGTGGGAAGAGACATTCGACGATGCGTACATCAAGCTGTATGAACTTTGGAAATCGAGAGGTCAATGACTATGTCAAACGTAAATACAGGTTCACTTAGTGTGGACAATaagaag                                                                                                                                                                                                                                                        <  1:44828/301‑1 (MQ=255)`  
                                                        `aCAGACTACGTGCTGGTAAGAAGTCAGAGTTAGCGTACTTAGAGAAAGACCTAGCGAAAGCAGACTTCTATAAAGAACTCTTTGAGAAACATAAGGATAAATGTTATGCATAACTTCAAGTCAACCCCACCTGCCGACAGCCTATCTGATGACTTCACATCTTGCTCAGAGTGGTGCCGAAAGATGTGGGAAGAGACATTCGACGATGCGTACATCAAGCTGTATGAACTTTGGAAATCGAGAGGTCAATGACTATGTCAAACGTATATACAGGTTCACTTAGTGTGGACAATAAGAAGtt                                                                                                                                                                                                                                                      >  1:227915/1‑301 (MQ=255)`  
                                                                 `gTGCTGGTAAGAAGTCAGAGTTAGCGTACTTAGAGAAAGACCTAGCGAAAGCAGACTTCTATAAAGAACTCTTTGAGAAACATAAGGATAAATGTTATGCATAACTTCAAGTCAACCCCACCTGCCGACAGCCTATCTGATGACTTCACATCTTGCTCAGAGTGGTGCCGAAAGATGTGGGAAGAGACATTCGACGATGCGTACATCAAGCTGTATGAACTTTGGAAAACGAGAGGTCAATGACTATGTCAAATGTAAATACAGGTTCACTTAGTGTGGACAATAAGAAGTTTTGGGCTAc                                                                                                                                                                                                                                             >  1:87150/1‑301 (MQ=255)`  
                                                                      `ggTAAGAAGTCAGAGTTAGCGTACTTAGAGAAAGACCTAGCGAAAGCAGACTTCTATAAAGAACTCTTTGAGAAACATAAGGATAAATGTTATGCATAACTTCAAGTCAACCCCACCTGCCGACAGCCTATCTGATGACTTCACATCTTGCTCAGAGTGGTGCCGAAAGATGTGGGAAGAGACATTCGACGATGCGTACATCAAGCTGTATGAACTTTGGAAATCGAGAGGTCAATGACTATGTCAAACGTAAATACAGGTTCACTTAGTGTGGTCAATAAGAAGGTTTGGGCTACCGTag                                                                                                                                                                                                                                        >  1:171947/1‑301 (MQ=255)`  
                                                                      `ggTAAGAAGTCAGAGTTAGCGTACTTAGAGAAAGACCTAGCGAAAGCAGACTTCTATAAAGAACTCTTTGAGAAACATAAGGATAAATGTTATGCATAACTTCAAGTCAACCCCACCTGCCGACAGCCTATCTGATGACTTCACATCTTGCTCAGAGTGGTGCCGAAAGATGTGGGAAGAGACATTCGACGATGCGTACATCAAGCTGTATGAACTTTGGAAATCGAGAGGTCAATGACTATGTCAAACGTAAATACAGGTTCACTTAGTGTGGACAATAAGAAGTTTTGGGCTACCGTag                                                                                                                                                                                                                                        <  1:109738/301‑1 (MQ=255)`  
                                                                        `tAAGAAGTCAGAGTTAGCGTACTGAGAGAAAGACCTAGCGAAAGCAGACTTCTATAAAGAACTCGTTGAGAAACATAAGGATAAATGTTATGCATAACTTCAAGTCAACCCCACCTGCCGACAGCCTATCTGATGACTTCACATCTTGCTCAGAGTGGTGCCGAAAGATGTGGGAAGAGACATTCGACGATGCGTACATCAAGCTGTATGAACTTTGGAAATCGAGAGGTCAATGACTATGTCAAACGTAAATACAGGTTCACTTAGTGTGGACAATAAGAAGTTTTGGGCTACCGTagag                                                                                                                                                                                                                                      <  1:180770/301‑1 (MQ=255)`  
                                                                                  `ggcttggagatgtgtaaaagagacagaGCCAAAGCAGACTTCTATAAATAACTCTTTGAGAAACATAAGGATAAATTTTATGCATAACTTCAAGTCAACCCCACCTGCCGACAACCTAGCCGATGACTTCACATCTTGCTCAGAGTGGTGCCGAAAGATTTTGGAAGAGACATTCGACGATGCGTACATCAAGCTGTATAAACTTTGGAAATCGAGAGGTCAATGACTATGTCAAACGTAAATACAGGTTCACTTAGTGTGGACAATAAGAAGTTTTGGGCTACCGTAGAGTCCTCGGAGc                                                                                                                                                                                                                            <  1:225624/275‑1 (MQ=255)`  
                                                                                      `ataggagccaggagaAAGACCTAGCAAAAGCAGACTTCAATAAAGAACTCTTGGAAAAAAATAAGGATAAATTTTAGGCAAAACTTCAAGTCAACCCCCCCTGCCGACAG‑CTATCTGATGACTTCACATCTTGCTCAGAGTGGTGCCGAAAGATGTGGGAAGAGACATTCGACGATGCGTACATCAAGCTGTATGAACTTTGGAAATCGAGAGGTCAATGACTATGTCAAACGTAAATACAGGTTCACTTAGTGTGGACAATAAGAAGTTTTGGGCTACCGTAGAGTCCTCGGAGCATTcc                                                                                                                                                                                                                       <  1:235503/290‑1 (MQ=255)`  
                                                                                       `agagTACTTAGAGAAAGACCTAGCGAAAGCAGACTTCTATAAAGAACTCTTTGAGAAACATAAGGATAAATGTTATGCATAACTTCAAGTCAACCCCACCTGCCGACAGACTATCTGATGACTTCACATCTTGATCAGAGTGGTGCCGAAAGATGTGGGAAGAGACATTCGACGATGCGTACATCAAGCTGTATGAACTTTGGAAATCGAGAGGTCAATGACTATGTCAAACGTAAATACAGGTTCACTTAGTGTGGACAATAAGAAGTTTTGGGCTACCGTAGAGTCCTCGGAGCATTcc                                                                                                                                                                                                                       <  1:154624/298‑1 (MQ=255)`  
                                                                                       `aGCGTACTTAGAGAAAGACCTAGCGAAAGCAGACTTCTATAAAGAACTCTTTGAGAAACATAAGGATAAATGTTATGCATAACTTCAAGTCAACCCCACCTGCCGACAGCCTATCTGATGACTTCACATCTTGCTCAGAGTGGTGCCGAAAGATGTGGGAAGAGACATTCGACGATGCGTACATCAAGCTGTATGAACTTTGGAAATCGAGAGGTCAATGACTATGTCAAACGTAAATACAGGTTCACTTAGTGTGGACAATAAGAAGTTTTGGGCTACCGTAGAGTCCTCGGAGCATTcc                                                                                                                                                                                                                       >  1:168448/1‑301 (MQ=255)`  
                                                                                                 `gagaAAGACCTAGCGAAAGCAGACTTCTATAAAGAACTCTTTGAGAAACATAAGGATAAATGTTATGCATAACTTCAAGTCAACCCCACCTGCCGACAGCCTATCTGATGACTTCACATCTTGCTCAGAGTGGTGCCGAAAGATGTGGGAAGAGACATTCGACGCTGCGTACATCAATCTGTATGTACTTTGGAATTCGAGAAGTCAATGACTATGCCAAACGTAAATACAGGTTCACTTAGTGTGGCCAATAAGTAGTTTTGGGTTAACGTAGAGTCCTCGGAGCATTACTTCGAGGttt                                                                                                                                                                                                             >  1:51573/1‑300 (MQ=255)`  
                                                                                                      `gatgtgtataagagacagcTTCTATAAAGAACACTTTGAGAAACATAAGGATAAATGTTATGCATAACTTCAAGTCAACCCCACCTGCCGACAGCCTATCTGATGACTTCACATCTTGCTCAGAGTGGTGCCGAAAGATGTGGGAAGAGACATTCGACGATGCGTACATCAAGCTGTATGAACTTTGGAAATCGAGAGGTCAATGACTATGTCAAACGTAAATACAGGTTCACTTAGTGTGGACAATAAGAAGTTTTGGGCTACCATAGAGTCCTCGGAGCATTCCTTCGAGGTTCCAATc                                                                                                                                                                                                        <  1:35558/283‑1 (MQ=255)`  
                                                                                                      `aGACCTAGCGAAAGCAGACTTCTATAAAGAACTCTTTGAGAAACATAAGGATAAATGTTATGCATAAATTCAAGTCAACCCCACCTGCAGACAGCCTATCTGATGACTTCACATCTTGCTCAGAGTGGTGCCGAAAGATGTGGGAAGAGACATTCGACGATGCGTACATCAAGCTGTATGAACTTTGGAAATCGAGAGGTCAATGACTATGTCAAACGTAAATACAGGTTCACTTAGTGGGGACAATAAGAAGTTTTGGGCTACCGTAGAGTCCTCGGAGCATTCCTTCGAGGTTCCAATc                                                                                                                                                                                                        <  1:143096/301‑1 (MQ=255)`  
                                                                                                           `gtataagagacagcgtCTATAAAGAACTCTTTGAGAAACATAAGGATAAATGTTATGCATAACTTCAAGTCAACCCCACCTGCCGACAGCCTATCTGATGACTTCACATCTTGCTCAGAGTGGTGCCGAAAGATGTGGGAAGAGACATTCGACGATGCGTACATCAAGCTGTATGAACTTTGGAAATCGAGAGGTCAATGACTATGTCAAACGTAAATACAGGTTCACTTAGTGTGGACAATAAGAAGTTTTGGGCTACCGTAGAGTCCTCGGAGCATTCCTTCGAGGTTCCAATCTACGc                                                                                                                                                                                                   <  1:57197/286‑1 (MQ=255)`  
                                                                                                            `aGCGAAAGCAGACTTCTATAAAGAACTCTTTGAGAAACATAAGGATAAATGTTATGCATAACTTCAAGTCAACCCCACCTGCCGACAGACTATCTGATGACTTCACATCTTGCTCAGAGTGGTGCCGAAAGATGTGGGAAGAGACATTCGACGATGCGTACATCAAGCTGTATGAACTTTGGAAATCGAGAGGTCAATGACTATGTCAAACGTAAATACAGGTTCACTTAGTGTGGACAATAAGAAGTTTTGGGCTACCGTAGAGTCCTCGGAGCATTCCTTCGAGGTTCCAATCTACGCt                                                                                                                                                                                                  <  1:97589/301‑1 (MQ=255)`  
                                                                                                                        `cTTCTATAAAGAACTCTTTGAGAAACATAAGGATAAATGTTATGCATAACTTCAAGTCAACCCCACCTGCCGACAGCCTATCTGATGACTTCACATCTTGCTCAGAGTGGTGCCGAAAGATGTGGGAAGAGACATTCGACGATGCGTACATCAAGCTGTATGAACTTTGGAAATCGAGAGGTCAATGACTATGTCAAACGTAAATACAGGTTCACTTAGTGTGGACAATAAGAAGTTTTGGGCTACCGTAGAGTCCTCGGAGCATTCCTTCGAGGTTCCAATCTACGCTGAGACCCTAGAc                                                                                                                                                                                      >  1:205369/1‑301 (MQ=255)`  
                                                                                                                          `tCTATAAAGAACTCTTTGAGAAACATAAGGATAAATGTTATGCATAACTTCAAGTCAACCCCACCTGCCGACAGCCTATCTGATGACTTCACATCTTGCTCAGAGTGGTGCCGAAAGATGTGGGAAGAGACATTCGACGATGCGTACATCAAGCTGTATGAACTTTTGAAATCGAGAGGTCAATGACTATGTCAAACGTAAATACAGGTTCACTTAGTGTGGACAATAAGAAGTTTTGGGCTACCGTAGAGTCCTCGGAGCATTCCTTCGCGGTTCCAATCTACGCTGAGAcctgtctctt                                                                                                                                                                                    >  1:178075/1‑293 (MQ=255)`  
                                                                                                                          `tCTATAAAGAACTCTTTGAGAAACATAAGGATAAATGTTATGCATAACTTCAAGTCAACCCCACCTGCCGACAGCCTATCTGATGACTTCACATCTTGCTCAGAGTGGTGCCGAAAGATGTGGGAAGAGACATTCGACGATGCGTACATCAAGCTGTATGAACTTTGGAAATCGAGAGGTCAATGACTATGTCAAACGTAAATACAGGTTCACTTAGTGTGGACAATAAGAAGTTTTGGGCTACCGTAGAGTCCTCGGAGCATTCCTTCGAGGTTCCAATCTACGCTGAGACCCTAGACGa                                                                                                                                                                                    >  1:16479/1‑301 (MQ=255)`  
                                                                                                                             `ataAAGAACTCTTTGAGAAACATAAGGATAAATGTTATGCATAACTTCAAGTCAACCCCACCTGCCGACAGCCTATCTGATGACTTCACATCTTGCTCAGAGTGGTGCCGAAAGATGTGGGAAGAGACATTCGACGATGCGTACATCAAGCTGTATGAACTTTGGAAATCGAGAGGTCAATGACTATGTCAAACGTAAATACAGGTTCACTTAGTGTGGACAATAAGAAGTTTTGGGCTACCGTAGAGTCCTCGGAGCATTCCTTCGAGGTTCCAATCTACGCTGAGACCCTAGACGAAGc                                                                                                                                                                                 >  1:264192/1‑301 (MQ=255)`  
                                                                                                                             `ataAAGAACTCTTTGAGAAACATAAGGATAAATGTTATGCATAACTTCAAGTCAACCCCACCTGCCGACAGCCTATCTGATGACTTCACATCTTGCTCAGAGTGGTGCCGAAAGATGTGGGAAGAGACATTCGACGATGCGTACATCAAGCTGTATGAACTTTGGAAATCGAGAGGTCAATGACTATGTCAAACGTAAATACAGGTTAACTTAGTGTGGACAATAAGAAGTTTTGGGCTACTGTAGAGTCCTCGGAGAATTCCTTCGAGGTTCCAATCTACGCTGAGACCCTAGACGAAGc                                                                                                                                                                                 >  1:305554/1‑301 (MQ=255)`  
                                                                                                                                  `gAACTCTTTGAGAAACATAAGGATTAATGTTATGCATAACTTCAAGTCAACCCCACCGGCCGACAGCCTTTCGTATGACTTCACATCTTGCTCAGAGTGGTGCCGAAAGATGTGGGAAGAGACATTCGAGGATGCGGACATCAAGCTGTATGAACTTTGGAAATCGAGAGGTCAATGACTATGTCAAACGTAAATACAGGTTCACTTAGTGTGGACAATAAGAAGTTTTGGGCTACCGTAGAGTCCTCGGAGCATTCCTTCGAGGTTCCAATCTACGCTGAGACCCTAGACGAAGCTCTgg                                                                                                                                                                            <  1:184224/301‑1 (MQ=255)`  
                                                                                                                                    `aCTCTTTGAGAAACATAAGGATAAATGTTATGCATAACTTCAAGTCAACCCCACCTGCCGACAGCCTATCTGATGACTTCACATCTTGCTCAGAGTGGTGCCGAAAGATGTGGGAAGAGACATTCGACGATGCGTACATCAAGCTGTATGAACTTTGGAAATCGAGAGGTCAATGACTATGTCAAACGTAAATACAGGTTCACTTAGTGTGGACAATAAGAAGTTTTGGGCTACCGTAGAGTCCTCGGAGCATTCCTTCGAGGTTCCAATCTACGCTGAGACCCTAGACGAAGCTCTGGAg                                                                                                                                                                          <  1:123116/301‑1 (MQ=255)`  
                                                                                                                                       `ctTTGAGAAACATAAGGATAAATGTTATGCATAACTTCAAGTCAACCCCACCTGCCGACAGCCTATCTGATGACTTCACATCTTGCTCAGAGTGGTGCCGAAAGATGTGGGAAGAGACATTCGACGATGCGTACATCAAGCTGTATGAACTTTGGAAATCGAGAGGTCAATGACTATGTCAAACGTAAATACAGGTTCACTTAGTGTGGACAATAAGAAGTTTTGGGCTACCGTAGAGTCCTCGGAGCATTCCTTCGAGGTTCCAATCTACGCTGAGACCCTAGACGAAGCTCTGGAGttt                                                                                                                                                                       >  1:24873/1‑300 (MQ=255)`  
                                                                                                                                              `aaaCATAAGGATAAATGTTATGCATAACTTCAAGTCAACCCCACCTTCCGACAGCCTATCTGATGACTTCACATCTTGCTCAGAGTGGTGCCGAAAGATGTGGGAAGAGACATTCGACGATGCGTACATCAAGCTGTATGAACTTTGGAAATCGAGAGGTCAATGACTATGTCAAACGTAAATACAGGTTCACTTAGTGTGGACAATAAGAAGTTTTGGGCTACCGTAGAGTCCTCGGAGCATTCCTTCGAGGTTCCAATCTACGCTGAGACCCTAGACGAAGCTCTGGAGTTAGCCGAAt                                                                                                                                                                >  1:202154/1‑301 (MQ=255)`  
                                                                                                                                                 `cATAAGGATAAATGTTATGCATAACTTCAAGTCAACCCCACCTGCCGACAGCCTATCTGATGACTTCACATCTTGCTCAGAGTGGTGCCGAAAGATGTGGGAAGAGACATTCGACGATGCGTACATCAAGCTGTATGAACTTTGGAAATCGAGAGGTCAATGACTATGTCAAACGTAAATACAGGTTCACTTAGTGTGGACAATAAGAAGTTTTGGGCTACCGTAGAGTCCTCGGAGCATTCCTTCGAGGTTCCAATCTACGCTGAGACCCTAGACGAAGCTCTGGAGTTAGCCGAATGGc                                                                                                                                                             >  1:237433/1‑301 (MQ=255)`  
                                                                                                                                                 `cATAAGGATAAATGTTATGCATAACTTCAAGTCAACCCCACCTGCCGACAGCCTATCTGATGAATTCACATCTTGCTCAGAGTGGTGACGAAAGATGTGGGAAGAGACATTCGACGATGCGTACATCAAGCTGTATGAACTTTGGAAATCGAGAGGTAAATGACTATGTCAAACGAAAATACAGGTTCACTTAGTGTGGACAATAAGAAGTTTTGGGCTAACGTAGAGTAATAGGATCATTCCTTCGAGGTTCCAATCTACGCTGAGACCCTAGAAGACGATCTGGAGGTAGcctgtatat                                                                                                                                                             >  1:316435/1‑294 (MQ=255)`  
                                                                                                                                                       `gATAAATGTTATGCATAACTTCAAGTCAACCCCACCTGCCGACAGCCTATCTGATGACTTCACATCTTGCTCAGAGTGGTGCCGAAAGATGTGGGAAGAGACATTCGACGATGCGTACATCAAGCTGTATGAACTTTGGAAATCGAGAGGTCAATGACTATGTCAAACGTAAATACAGGTTCACTTAGTGTGGACAATAAGAAGTTTTGGGCTACCGTAGAGTCCTCGGAGCATTCCTTCGAGGTTCCAATCTACGCTGAGACCCTAGACGAAGCTCTGGAGTTAGCCGAATGGCAATAcc                                                                                                                                                       >  1:130037/1‑300 (MQ=255)`  
                                                                                                                                                       `gATAAATGTTATGCATAACTTCAAGTCAACCCCACCTGCCGACAGCCTATCTGATGACTTCACATCTTGCTCAGAGTGGTGCCGAAAGATGTGGGAAGAGACATTCGACGATGCGTACATCAAGCTGTATGAACTTTGGAAATCGAGAGGTCAATGACTATGTCAAACGTAAATACAGGTTCACTTAGTGTGGACAATAAGAAGTTTTGGGCTACCGTAGAGTCCTCGGAGCATTCCTTCGAGGTTCCAATCTACGCTGAGACCCTAGACGAAGCTCTGGAGTTAGCCGAATGGCAATACg                                                                                                                                                       >  1:186840/1‑301 (MQ=255)`  
                                                                                                                                                           `aaTGTTATGCATAACTTCAAGTCAACCCCACCTGCCGACAGCCTATCTGATGACGTCACATCTTGCTCAGAGTGGTGCCGAAAGATGTGGGAAGAGACATTCGACGATGCGTACATCAAGCTGTATGAACGTTGGAAATCGAGAGGTCAATGACTATGTCAAACGTAAATACAGGTTCACTTAGTGTGGACAATAAGAAGTTTTGGGCTACCGTAGAGTCCTCGGAGCATTCCTTCGAGGTTCCAATCTACGCTGAGACCCTAGACGAAGATCTGGAGTTAGCCGAATGGCAATACGTTcc                                                                                                                                                   <  1:259286/301‑1 (MQ=255)`  
                                                                                                                                                            `aTGTTATGCATAACTTCAAGTCAACCCCACCTGCCGACAGCCTATCTGATGACTTCACATCTTGCTCAGAGTGGTGCCGAAAGATGTGGGAAGAGACATTCGACGATGCGTACATCAAGCTGTATGAACTTTGGAAATCTAGAGGTCAATGACTATGTCAAACGTAAATACAGGTTCACTTAGTGTGGACAATAAGAAGTTTTGGGCTACCGTAGAGTCCTCGGAGCATTCCTTCGAGGTTCCAATCTACGCTGAGACCCTAGACGAAGCTCTGGAGTTAGCCGAATGGctgtctcttatt                                                                                                                                                  >  1:294934/1‑290 (MQ=255)`  
                                                                                                                                                            `aTGTTATGCATAACTTCAAGTCAACCCCACCTGCCGACAGCCTATCTGATGACTTCACATCTTGCTCAGAGTGGTGCCGAAAGATGTGGGAAGAGACATTCGACGATGCGTACATCAAGCTGTATGAACTTTGGAAATCGAGAGGTCAATGACTATGTCAAACGTAAATACAGGTTCACTTAGTGTGGACAATAAGAAGTTTTGGGCTACCGTAGAGTCCTCGGAGCATTCCTTCGAGGTTCCAATCTACGCTGAGACCCTAGACGAAGCTCTGGAGTTAGCCGAATGGCAATACGTTCCg                                                                                                                                                  >  1:157203/1‑301 (MQ=255)`  
                                                                                                                                                                   `gCATAACTTCAAGTCAACCCCACCTGCCGACAGCCTATCTGATGACTTCACATCTTGCTCAGAGTGGTGCCGAAAGATGTGGGAAGAGACATTCGACGATGCGTACATCAAGCTGTATGAACTTTGGAAATCGAGAGGTCAATGACTATGTCAAACGTAAATACAGGTTCACTTAGTGTGGACAATAAGAAGTTTTGGGCTACCGTAGAGTCCTCGGAGCATTCCTTCGAGGTTCCAATCTACGCTGAGACCCTAGACGAAGCTCTGGAGTTAGCCGAATGGCAATACgctgtctcttata                                                                                                                                           >  1:256171/1‑289 (MQ=255)`  
                                                                                                                                                                   `gCATAACTTCAAGTCAACCCCACCTGCCGACAGCCTATCTGATGACTTCACATCTTGCTCAGAGTGGTGCCGAAAGATGTGGGAAGAGACATTCGACGATGCGTACATCAAGCTGTATGAACTTTGGAAATCGAGAGGTCAATGACTATGTCAAACGTAAATACAGGTTCACTTAGTGTGGACAATAAGAAGTTTTGGGCTACCGTAGAGTCCTCGGAGCATTCCTTCGAGGTTCCAATCTACGCTGAGACCCTAGACGAAGCTCTGGAGTTAGCCGAATGGCAATACGTTCCggctggct                                                                                                                                           >  1:47676/1‑301 (MQ=255)`  
                                                                                                                                                                        `aCTTCAAGTCAACCCCACCTGCCGACAGCCTATCTGATGACTTCACATCTTGCTCAGAGTGGTGCCGAAAGATGTGGGAAGAGACATTCGACGATGCGTACATCAAGCTGTATGAACTTTGGAAATCGAGAGGTCAATGACTATGTCAAACGTAAATACAGGTTCACTTAGTGTGGACAATAAGAAGTTTTGGGCTACCGTAGAGTCCTCGGAGCATTCCTTCGAGGTTCCAATCTACGCTGAGACCCTAGACGAAGCTCTGGAGTTATCCGAATGGCAATACGTTCCGGCTGGCTTTGAg                                                                                                                                      >  1:116588/1‑301 (MQ=255)`  
                                                                                                                                                                         `cTTCAAGTCAACCCCACCTGCCGACAGCCTATCTGATGACTTCACATCTTGCTCAGAGTGGTGCCGAAAGATGTGGGAAGAGACATTCGACGATGCGTACATCAAGCTGTATGAACTTTGGAAATCGAGAGGTCAATGACTATGTCAAACGTAAATACAGGTTCACTTAGTGTGGACAATAAGAAGTTTTGGGCTACCGTAGAGTCCTCGGAGCATTCCTTCGAGGTTCCAATCTACGCTGAGACCCTAGACGAAGCTCTGGAGTTAGCCGAATGGCAATACGTTCCGGCTGGCTTTGAgg                                                                                                                                     <  1:70156/301‑1 (MQ=255)`  
                                                                                                                                                                         `cTTCAAGTCAACCCCACCTGCCGACAGCCTATCTGATGACTTCACATCTTGCTCAGAGTGGTGCCGAAAGATGTGGGAAGAGACATTCGACGATGCGTACATCAAGCTGTATGAACTTTGGAAATCGAGAGGTCAATGACTATGTCAAACGTAAATACAGGTTCACTTAGTGTGGACAATAAGAAGTTTTGGGCTACCGTAGAGTCCTCGGAGCATTCCTTCGAGGTTCCAATCTACGCTGAGACCCTAGACGAAGCTCTGGAGTTAGCCGAATGGCAATACGTTCCGGCTGGCTTTGAgg                                                                                                                                     >  1:24172/1‑301 (MQ=255)`  
                                                                                                                                                                         `cTTCAAGTCAACCCCACCTGCCGACAGCCTATCTGATGACTTCACATCTTGCTCAGAGTGGTGCCGAAAGATGTGGGAAGAGACATTCGACGATGCGTACATCAAGCTGTATGAACTTTGGAAATCGAGAGGTCAATGACTATGTCAAACGTAAATACAGGTTCACTTAGTGTGGACAATAAGAAGTTTTGGGCTACCGTAGAGTCCTCGGAGCATTCCTTCGAGGTTCCAATCTACGCTGAGACCCTAGACGAAGCTCTGGAGTTAGCCGAATGGCAATACGTTCCGGCTGGCTTTAAgg                                                                                                                                     >  1:323923/1‑301 (MQ=255)`  
                                                                                                                                                                         `cTTCAAGTCAACCCCACCTGCCGACAGCCTATCTGATGACTTCACATCTTGCTCAGAGTGGTGCCGAAAGATGTGGGAAGAGACATTCGACGATGCGTACATCAAGCTGTATGAACTTTGGAAATCGAGAGGTCAATGACTATGTCAAACGTAAATACAGGTTCAATTAGTGTGGACAATAAGAAGTTTTGGGCTACCGTAGAGTCCTCGGAGCATTCCTTCGAGGTTCCAATCTACGCTGAGACCCTAGACGAAGCTCTGGAGTTAGCCGAATGGCAATACGtctgtctcttatacacat                                                                                                                                     >  1:196515/1‑284 (MQ=255)`  
                                                                                                                                                                              `aGTCAACCCCACCTGCCGACAGCCTATCTGATGACTTCACTTCTTGCTCAGAGTGGTGCCGAAAGATGTGGGAAGAGACATTCGACGATGCGTACATCAAGCTGTATGAACTTTGGAAATCGAGAGGTCAATGACTATGTCAAACGTAAATACAGGTTCACTTAGTGTGGACAATAAGAAGTTTTGGGCTACCGTAGAGTCCTCGGAGCATTCCTTCGAGGTTCCAGTCTACGCTGAGACCCTAGACGAAGCTCTGGAGTTAGCCGAATGGCAATACGTCCCGGCTGGCTTTGAGGTTACt                                                                                                                                >  1:223433/1‑301 (MQ=255)`  
                                                                                                                                                                                      `ccACCTGCCGACAGCCTATCTGATGACTTCACATCTTGCTCAGAGTGGTGCCGAAAGATGTGGGAAGAGACATTCGACGATGCGTACATCAAGCTGTATGAACTTTGGAAATCGAGAGGTCAATGACTATGTCAAACGTAAATACAGGTTCACTTAGTGTGGACAATAAGAAGTTTTGGGCTACCGTAGAGTCCTCGGAGCATTCCTTCGAGGTTCCAATCTACGCTGAGACCCTAGACGAAGCTCTGGAGTTAGCCGAATGGCAATACGTTCCGGCTGGCTTTGAGATTACTCGTGTGCg                                                                                                                        >  1:138774/1‑301 (MQ=255)`  
                                                                                                                                                                                       `cACCTGCCGACAGCCTATCTGATGACTTCACATCTTGCTCAGAGTGGTGCCGAAAGATGTGGGAAGAGACATTCGACGATGCGTACATCAAGCTGTATGAACTTTGGAAATCGAGAGGTCAATGACTATGTCAAACGTAAATACAGGTTCACTTAGTGTGGACAATAAGAAGTTTTGGGCTACCGTAGAGTCCTCGGAGCATTCCTTCGAGGTTCCAATCTACGCTGAGACCCTAGACGAAGCTCTGGAGTTAGCCGAATGGCAATACGTTCCGGCTGGCTTTGAGGTTACTCGTGTTCGt                                                                                                                       >  1:1745/1‑301 (MQ=255)`  
                                                                                                                                                                                         `ccTGCCGACAGCCTATCTGATGACTTCACATCTTGCTCAGAGTGGTGCCGAAAGATGTGGGAAGAGACATTCGACGATGCGTACATCAAGCTGTATGAACTTTGGAAATCGAGAGGTCAATGACTATGTCAAACGTAAATACAGGTTCACTTAGTGTGGACAATAAGAAGTTTTGGGCTACCGTAGAGTCCTCGGAGCATTCCTTCGAGGTTCCAATCTACGCTGAGACCCTAGACGAAGCTCTGGAGTTAGCCGAATGGCAATACGTTCCGGCTGGCTTTGAGGTTACTCGTGTGCGTcc                                                                                                                     <  1:39164/301‑1 (MQ=255)`  
                                                                                                                                                                                            `gCCGACAGCCTATCTGATGACTTCACATCTTGCTCAGAGTGGTGCCGAAAGATGTGGGAAGAGACATTCGACGATGCGTACATCAAGCTGTATGAACTTTGGAAATCGAGAGGTCAATGACTATGTCAAACGTAAATACAGGTTCACTTAGTGTGGACAATAAGAAGTTTTGGGCTACCGTAGAGTCCTCGGAGCATTCCTTCGAGGTTCCAATCTACGCTGAGACCCTAGCCGAAGCTCTGGAGTTAGCCGAATGGCAATACGTTCCGGCTGGCTTAGAGGTTACTCGTGTGCGTCCTtg                                                                                                                  >  1:111980/1‑301 (MQ=255)`  
                                                                                                                                                                                               `gACAGCCTATCTGATTACTTCACATCTTGCTCAGAGTGGTGCCGAAAGATGTGGGAAGAGACATTCGACGATGCGTACATCAAGCTGTATGAACTTTGGAAATCGAGAGGTCAATGACTATGTCAAACGTAAATACAGGTTCACTTAGTGTGGACAATAAGAAGTTTTGGGCTACCGTAGAGTCCTCGGAGCATTCCTTCGAGGTTCCAATCTACGCTGAGACCCTAGACGAAGCTCTGGAGTTAGCCGAATGGCAATACGTTCCGGCTGGCTTTGAGGTTACTCGTGTGCGTCCTTgtgt                                                                                                               <  1:312379/301‑1 (MQ=255)`  
                                                                                                                                                                                                   `gCCTATCTGATGACTTCACATCTTGCTCAGAGTGGTGCCGAAAGATGTGGGAAGAGACATTCGACGATGCGTACATCAAGCTGTATGAACTTTGGAAATCGAGAGGTCAATGACTATGTCAAACGTAAATACAGGTTCACTTAGTGTGGACAATAAGAAGTTTTGGGCTACCGTAGAGTCCTCGGAGCATTCCTTCGAGGTTCCAATCTACGCTGAGACCCTAGACGAAGCTCTGGAGTTAGCCGAATGGCAATACGTTCCGGCTGGCTTTGAGGTTCCTCGTGTGCGTCCTTGTGTAGcc                                                                                                           >  1:297654/1‑300 (MQ=255)`  
                                                                                                                                                                                                   `gCCTATCTGATGACTTCACATCTTGCTCAGAGTGGTGCCGAAAGATGTGGGAAGAGACATTCGACGATGCGTACATCAAGCTGTATGAACTTTGGAAATCGAGAGGTCAATGACTATGTCAAACGTAAATACAGGTTCACTTAGTGTGGACAATAAGAAGTTTTGGGCTACCGTAGAGTCCTCGGAGCATTCCTTCGAGGTTCCAATCTACACTGAGACCCTAGACGAAGCTCTGGAGTTAGCCGAATGGCAATACGTTCCGGCTGGCTTTGAGGTTACTCGTGTTCGTCCTTGTTTAGcc                                                                                                           >  1:206889/1‑300 (MQ=255)`  
                                                                                                                                                                                                    `tataagagacagcatCACATCTTGCTCAGAGTGGTGCCGAAAGATGTGGGAAGAGACATTCGACGATGCGTACATCAAGCTGTATGAACTTTGGAAATCGAGAGGTCAATGACTATGTCAAACGTAAATACAGGTTCACTTAGTGTGGACAATAAGAAGTTTTGGGCTACCGTAGAGTCCTCGGAGCATTCCTTCGAGGTTCCAATCTACGCTGAGACCCTAGACGAAGCTCTGGAGTTAGCCGAATGGCAATACGTTCCGGCTGGCTTTGAGGTTACTCGTGTGCGTCCTTGTGTAGCAc                                                                                                          <  1:219976/287‑1 (MQ=255)`  
                                                                                                                                                                                                    `ccTATCTGATGACTTCACATCTTGCTCAGAGTGGTGCCGAAAGATGTGGGAAGAGACATTCGACGATGCGTACATCAAGCGGTATGAACGTTGGAAATCGAGAGGTCAATGACTATGTCAAACGTAAATACAGGTTCACTTAGTGTGGACAATAAGAAGTTTTGGGCTACCGTAGAGTCCTCGGAGCATTCCTTCGAGGTTCCAATCTACGCTGAGACCCTAGACGAAGCTCTGGAGTTAGCCGAATGGCAATACGTTCCGGCTGGCTTTGAGGTTACTCGTGTGCGTCCTTGTGTAGCAc                                                                                                          <  1:160057/301‑1 (MQ=255)`  
                                                                                                                                                                                                    `acagtCTGATGACTTCACATCTTGCTCAGAGTGGTGCCGAAAGATGTGGGAAGAGACATTCGACGATGCGTAAATCAAGCTGTATGAACTTTGGAAATCGAGAGGTCAATGACTATGTCAAACGTAAATACAGGTTCACTTAGTGTGGACAATAAGAAGTTTTGGGCTACCGTAGAGTCCTCGGAGCATTCCTTCGAGGTTCCAATCTACGCTGAGACCCTAGACGAAGCTCTGGAGTTAGCCGAATGGCAATACGTTCCGGCTGGCTTTGAGGTTACTCGTGTGCGTCCTTGTGTAGCAc                                                                                                          <  1:329840/297‑1 (MQ=255)`  
                                                                                                                                                                                                     `cTATCTGATGACTTCACATCTTGCTCAGAGTGGTGCCGAAAGATGTGGGAAGAGACATTCGACGATGCGTACATCAAGCTGTATGAACTTTGGAAATCGAGAGGTCAATGACTATGTCAAACGTAAATACAGGTTCACTTAGTGTGGACAATAAGAAGTTTTGGGCTACCGTAGAGTCCTCGGAGCATTCCTTCGAGGTTCCAATCTACGCTGAGACCCTAGACGAAGCTCTGGAGTTAGCCGAATGGCAATACGTTCCGGCTGGCTTTGAGGTTACTCGTGTGCGTCCTTGTGTAGCAcc                                                                                                         >  1:103877/1‑301 (MQ=255)`  
                                                                                                                                                                                                        `tCTGATGACTTCACATCTTGCTCAGAGTGGTGCCGAAAGATGTGGGAAGAGACATTCGACGATGCGTACATCAAGCTGTATGAACTTTGGAAATCGAGAGGTCAATGACTATGTCAAACGTAAATACAGGTTCACTTAGTGTGGACAATACGAAGTTTTGGGCTACCGTAGAGTCCTCGGAGCATTCCTTCGAGGTTCCAATCTACGCTGAGACCCTAGACGAAGCTCTGGAGTTAGCCGAATGGCAATACGTTCCGGCTGGCTTTGAGGTTACTCGTGTGCGTCCTTTTGTAGCACCGaa                                                                                                      >  1:189116/1‑301 (MQ=255)`  
                                                                                                                                                                                                        `tCTGATGACTTCACATCTTGCTCAGAGTGGTGCCGAAAGATGTGGGAAGAGACATTCGACGATGCGTACATCAAGCTGTATGAACTTTGGAAATCGAGAGGTCAATGACTATGTCAAACGTAAATACAGGTTCACTTAGTGTGGACAATAAGAAGTTTTGGGCTACCGTAGAGTCCTCGGAGCATTCCTTCGAGGTTCCAATCTACGCTGAGACCCTAGACGAAGCTCTGGAGTTAGCCGAATGGCAATACGTTCCGGCTGGCTTTGAGGTTACTCGTGTGCGTCCTTGTGTAGCACCGaa                                                                                                      >  1:33829/1‑301 (MQ=255)`  
                                                                                                                                                                                                              `gaCTTCACATCTTGCTCAGAGTGGTGCCGAAAGATGTGGGAAGAGACATTCGACGATGCGTACATCAAGCTGTATGAACTTTGGAAATCGAGAGGTCAATGACTATGTCAAACGTAAATACAGGTTCACTTAGTGTGGACAATAAGAAGTTTTGGGCTACCGTAGAGTCCTCGGAGCATTCCTTCGAGGTTCCAATCTACGCTGAGACCCTAGACGAAGCTCTGGAGTTAGCCGAATGGCAATACGTTCCGGCTGGCTTTGAGGTTACTCGTGTGCGTCCTTGTGTAGCACCGAAGTAATa                                                                                                >  1:210528/1‑301 (MQ=255)`  
                                                                                                                                                                                                               `gcTTCACAGCTTGCTCAGAGTGGTGCCGAAAGATGTGGGAAGAGACATTCGACGATGCGTACATCAAGCTGTATGAACTTTGGAAATCGAGAGGTCAATGACTATGTCAAACGTAAATACAGGTTCACTTAGTGTGGACAATAAGAAGTTTTGGGCTACCGTAGAGTCCTCGGAGCATTCCTTCGAGGTTCCAATCTACGCTGAGACCCTAGACGAAGCTCTGGAGTTAGCCGAATGGCAATACGTTCCGGCTGGCTTTGAGGTTACTCGTGTGCGTCCTTGTGTAGCACCGAAGTAATAc                                                                                               <  1:92894/300‑1 (MQ=255)`  
                                                                                                                                                                                                               `aCTTCACATCTTGCTCAGAGTGGTGCCGAAAGATGTGGGAAGAGACATTCGACGATGCGTACATCAAGCTGTATGAACTTTGGAAATCGAGAGGTCAATGACTATGTCAAACGTAAATACAGGTTCACTTAGTGTGGACAATAAGAAGTTTTGGGCTACCGTAGAGTCCTCGGAGCATTCCTTCGAGGTTCCAATCTACGCTGAGACCCTAGACGAAGCTCTGGAGTTAGCCGAATGGCAATACGTTCCGGCTGGCTTTGAGGTTACTCGTGTGCGTCCTTGTGTAGCACCGAAGTAATAc                                                                                               <  1:278620/301‑1 (MQ=255)`  
                                                                                                                                                                                                                      `aTCTTGCTCAGAGTGGTGCCGAAAGATGTGGGAAGAGACATTCGACGATGCGTACATCAAGCTGTATGAACGTTGGAAATCGAGAGGTCAATGACTATGTCAAACGTAAATACAGGTTCACTTAGTGTGGACAATAAGAAGTTTTGGGCTACCGTAGAGTCGTCGGAGCATTCCTTCGAGGTTCCAATCTACGCTGAGACCCTAGACGAAGCTCTGGAGTTAGCCGAATGGCAATACGTTCCGGCTGGCTTTGAGGTTACTCGTGTGCGTCCTTGTGTAGCCCCGAAGTAATACGACTCat                                                                                        >  1:59652/1‑300 (MQ=255)`  
                                                                                                                                                                                                                           `gCTCAGAGTGGTGCCGAAAGATGTGGGAAGAGACATTCGACGATGCGTACATCAAGCTGTATGAACTTTGGAAATCGAGAGGTCAATGACTATGTCAAACTTAAATACAGGTTCACTTAGTGAGGACAATCAGAAGGTTTGGGCTACCGTAGAGTCCTCGGAGCATTCCTACGCTGTTCCAATCTACGCTGAGATCCTAGACGAAGCTCTGGAGTTCGCCGATTGGCCATACGTTCCGGCCGGCTTTGAGGTTACTCGTGTGCGTCCTTATCTCGCACCTCAGTCATAGGACTCACTTTTa                                                                                   >  1:332175/1‑301 (MQ=255)`  
                                                                                                                                                                                                                           `gCTCAGAGTGGTGCCGAAAGATGTGGGAAGAGACATTCGACGATGCGTACATCAAGCTGTATGAACTTTGGAAATCGAGAGGTCAATGACTATGTCAAACGTAAATACAGGTTCACTTAGTGTGGACAATAAGAAGTTTTGGGCTACCGTAGAGTCCTCGGAGCATTCCTTCGAGGTTCCAATCTACGCTGAGACCCTAGACGAAGCTCTGGAGTTAGCCGAATGGCAATACGTTCCGGCTGGCTTTGAGGTTACTCGTGTGCGTCCTTGTGTAGCACCGAAGTAATACGACTCACTATTa                                                                                   >  1:261410/1‑301 (MQ=255)`  
                                                                                                                                                                                                                           `gCTCAGAGTGGTGCCGAAAGATGTGGGAAGAGACATTCGACGATGCGTACATCAAGCTGTATGAACCTTGGAAATCGAGAGGTCAATGACTATGTCAAACGTAAATACAGGTTCACTTAGTGTGGACAATAAGAAGTTTTGGGCTACCGTAGAGTCCTCGGAGCATTCCTTCGAGGTTCCAATCTACGCTGAGACCCTAGACGAAGCTCTGGAGTTAGCCGAATGGCAATACGTTCCGGCTGGCTTTGAGGTTACTCGTGTGCGTCCTTGTGTAGCAcctgtctcttatacacatctccgc                                                                                   >  1:274284/1‑279 (MQ=255)`  
                                                                                                                                                                                                                              `cAGAGTGGTGCCGAAAGATGTGGGAAGAGACATTCGACGATGCGTACATCAAGCTGTATGAACTTTGGAAATCGAGAGGTCAATGACTATGTCAAACGTAAATACAGGTTCACTTAGTGTGGACAATAAGAAGTTTTGGGCTACCGTAGAGTCCTCGGAGCATTCCTTCGAGGTTCCAATCTACGCTGAGACCCTAGACGAAGCTCTGGAGTTAGCCGAATGGCAATACGTTCCGGCTGGCTTTGAGGTTACTCGTGTGCGTCCTTGTGTAGCACCGAAGTAATACGACTCACTATTAggg                                                                                <  1:308947/301‑1 (MQ=255)`  
                                                                                                                                                                                                                              `cAGAGTGGTGCCGAAAGAGGTGGGAAGAGACATTCGACGATGCGTACATCAAGCTGTATGAACTTTGGAAATCGAGAGGTCAATGACTATGTCAAACGTAAATACAGGTTCACTTAGTGTGGACAATAAGAAGTTTTGGGCTACCGTAGAGTCCTCGGAGCATTCCTTCGAGGTTCCAATCTACGCTGAGACCCTAGACGAAGCTCTGGAGTTAGCCGAATGGCAATACGTTCCGGCTGGCTTTGAGGTTACTCGTGTGCGTCCTTGTGTAGCACCGAAGTAATACGACTCACTATTAggg                                                                                <  1:84927/301‑1 (MQ=255)`  
                                                                                                                                                                                                                              `aagagTGGTGCCGAAAGAGGTGGGAAGAGACATTCGACGATGCGTACATCAAGCTGTATGAACTTTGGAAATCGAGAGGTCAATGACTATGTCAAACGTAAATACAGGTTCACTTAGTGTGGACAATAAGAAGTTTTGGGCTACCGTAGAGTCCTCGGAGCATTCCTTCGAGGTTCCAATCTACGCTGAGACCCTAGACGAAGCTCTGGAGTTAGCCGAATGGCAATACGTTCCGGCTGGCTTTGAGGTTACTCGTGTGCGTCCTTGTGTAGCACCGAAGTAATACGACTCACTATTAggg                                                                                <  1:94668/300‑1 (MQ=255)`  
                                                                                                                                                                                                                                `gagTGGTGCCGAAAGATGTGGGAAGAGACATTCGACGATGCGTACATCAAGCTGTATGAACTTTGGAAATCGAGAGGTCAATGACTATGTCAAACGTAAATACAGGTTCACTTAGTGTGGACAATAAGAAGTTTTGGGCTACCGTAGAGTCCTCGGAGCATTCCTTCGAGGTTCCAATCTACGCTGAGACCCTAGACGAAGCTCTGGAGTTAGCCGAATGGCAATACGTTCCGGCTGGCTTTGAGGTTACTCGTGTGCGTCCTTGTGTAGCACCGAAGTAATACGACTCACTATTAgggct                                                                              >  1:268104/1‑299 (MQ=255)`  
                                                                                                                                                                                                                                   `tggtgCCGAAAGATGTGGGAAGAGACATTCGACGATGCGTACATCAAGCTGTATGAACTTTGGAAATCGAGAGGTCAATGACTATGTCAAACGTAAATACAGGTTCACTTAGTGTGGACAATAAGAAGTTTTGGGCTACCGTAGAGTCCTCGGAGCATTCCTTTGAGGTTCCAATCTACGCTGAGACCCTAGACGAAGCTCTGGAGTTAGCCGAATGGCAATACGTTCCGGCTGGCTTTGAGGTTACTCGTGTGCGTCCTTGTGTAGCACCGAAGTAATACGACTCACTATTAGGGAAGAc                                                                           <  1:147004/301‑1 (MQ=255)`  
                                                                                                                                                                                                                                         `caaagaaTGGGGGAAGAGACATTAGACGATGCGTACATCAAGCTGTATGAACTTTGGAAATCGAGAGGTCAATGACTATGTCAAACGTAAATACAGGTTCACTTAGTGTGGACAATAAGAAGTTTTGTGCTACCGTAGAGTCCTCGGAGCATTCCTTCGAGGTTCCAATCTACGCTGAGACCCTAGACGAAGCTCTGGAGTTAGCCGAATGGCAATACGTTCCGGCTGGCTTTGAGGTTACTCGTGTGCGTCCTTGTGTAGCACCGAAGTAATACGACTCACTATTAGGGAAGACTCCctc                                                                     <  1:320684/295‑1 (MQ=255)`  
                                                                                                                                                                                                                                                           `aCATTCGACGATGCGTACATCAAGCTGTATGAACTTTGGAAATCGAGAGGTCAATGACTATGTCAAACGTAAATACAGGTTCACTTAGTGTGGACAATAAGAAGTTTTGGGCTACCGTAGAGTCCTCGGAGCATTCCTTCGAGGTTCCAATCTACGCTGAGACCCTAGACGAAGCTCTGGAGTTAGCCGAATGGCAATACGTTCCGGCTGGCTTTGAGGTTACTCGTGTGCGTCCTTGTGTAGCACCGAAGTAATACGACTCACTATTAgggctgtctcttatacacatctccgagcccac                                                   >  1:12679/1‑272 (MQ=255)`  
                                                                                                                                                                                                                                                                  `acgaTGCGTACATCAAGCTGTATGAACTTTGGAAATCGATAGGTCAATGACTATGTCAAACGTAAATACAGGTTCACTTAGTGTGGACAATAAGAAGTTTTGGGCTACCGTAGAGTCCTCGGAGCATTCCTTCGAGGTTCCAATCTACGCTGAGACCCTAGACGAAGCTCTGGAGTTAGCCGAATGGCAATACGTTCCGGCTGGCTTTGAGGTTACTCGTGTGCGTCCTTGTGTAGCACCGAAGTAATACGACTCACTATTAGGGAAGACTCCCTCTGAGAAACCAAACGAAACCTAAAgg                                            <  1:283043/301‑1 (MQ=255)`  
                                                                                                                                                                                                                                                                            `cATCAAGCTGTATGAACTTTGGAAATCGAGAGGTCAATGACTATGTCAAACGTAAATACATGTTCACTTAGTGTGGACAATAAGAAGTTTTGGGCTACCGTAGAGTCCTCGGAGCATTCCTTCGAGGTTCCAATCTACGCTGAGACCCTAGGCGAAGCTCTGGAGTTAGCCGAATGGCAATACGTTCCGGCTGGCTTTGAGGTTACTCGTGTGCGTCCTTGTGTAGCACCGAAGTAATACGACTCACTATTAGGGAAGACTCCCTCTGAGAAACCAAACGAAACCTAAAGGAGATTAACAt                                  <  1:42696/301‑1 (MQ=255)`  
                                                                                                                                                                                                                                                                            `aataaaGCTGTATGAACTTTGGAAATCGAGAGGTCAATGACTATGTAAAACGTAAATACAGGTTCACTTAGTGTGGACAATAAGAAGTTTTGGGCTACCGTAGAGTCCTAGGAGCATTCCTTCGAGGTTCCAATCTACGATGAGACCCTAGACGAAGCTCTGGAGTTAGCCGAATGGCAATACGTTCCGGCTGGCTTTGAGGTTACTCGTGTGCGTCCTTGTGTAGCACCGAAGTAATACGACTCACTATTAGGGAAGACTCCCTCTGAGAAACCAAACGAAACCTAAAGGAGATTAACAt                                  <  1:66995/297‑1 (MQ=255)`  
                                                                                                                                                                                                                                                                                 `acaggTATGAACTTTGGAAATCGAGAGGTCAATGACTATGTCAAACGTAAATACAGGTTCACTTAGTGTGGACAATAAGAAGTTTTGGGCTACCGTAGAGTCCTCGGAGCATTCCTTCGAGGTTCCAATCTACGCTGAGACCCTAGACGAAGCTCTGGAGTTAGCCGAATGGCAATACGTTCCGGCTGGCTTTGAGGTTACTCGTGTGCGTCCTTGTGTAGCACCGAAGTAATACGACTCACTATTAGGGAAGACTCCCTCTGAGAAACCAAACGAAACCTAAAGGAGATTAACATTATgg                             <  1:100215/297‑1 (MQ=255)`  
                                                                                                                                                                                                                                                                                     `gTATGAACTTTGGAAATCGAGAGGTCAATGACTATGTCAAACGTAAATACAGGTTCACTTAGTGTGGACAATAATAAGTTTTGGGCTACCGTAGAGTCCTCGTAGCATTCCTTCGATGTTCCAATCTACGATGAGACCCTAGACGAAGCTCTGGAGTTAGCCGAATTGCAATACGTTCCGGCTGGCTTTGAGGTTACTCGTGTGCGTCCTTGTGTAGCACCGAAGTAATACGACTCACTATTAGGGACGACTCCCTCTGAGAAACCAAACGCAACCTAACTGAGATTAACATTATgtataa                         >  1:29968/1‑296 (MQ=255)`  
                                                                                                                                                                                                                                                                                     `gTATGAACTTTGGAAATCGAGAGGTCAATGACTATGTCAAACGTAAATACAGGTTCACTTAGTGTGGACAATAAGAAGTTTTGGGCTACCGTAGAGTCCTCGGAGCATTCCTTCGAGGTTCCAATCTACGCTGAGACCCTAGACGAAGCTCTGGAGTTAGCCGAATGGCAATACGTTCCGGCTGGCTTTGAGGTTACTCGTGTGCGTCCTTGTTTAGTACCGAAGTAATACGACTCACTATTAGGGAAGACTCCCTCTGAGAAACCAAACGAAACCTAAAGGAGATTAACATTATGGCTaa                         >  1:82131/1‑301 (MQ=255)`  
                                                                                                                                                                                                                                                                                                    `aTCGAGAGGTCAATGACTATGTCAAACGTAAATACAGGTTCACTTAGTGTGGACAATAAGAAGGTTTGGGCTACCGTAGAGTCCTCGGAGCATTCCTTCGAGATTCCAATCTACGCTGAGACCCTAGACGAAGCTCTGGAGTTAGCCGAAAGGCAATACGTTCCGGCTGGCTTTGAGGTTACTCGTGTGCGTCCTTGTGTAGCACCGAAGTAATACGACTCACTATTAGGGAAGACTCCCTCTGAGAAACCAAACGAAACCTAAAGGAGATTAACATTATGGCTAAGAAGATTTTCACctc          <  1:327020/301‑1 (MQ=255)`  
                                                                                                                                                                                                                                                                                                         `gagGTCAATGACTATGTCAAACGTAAATACAGGTTCACTTAGTGTGGACAATAAGAAGTTTTGGGCTACCGTAGAGTCCTCGGAGCATTCCTTCGAGGTTCCAATCTACGCTGAGACCCTAGACGAAGCTCTGGAGTTAGCCGAATGGCAATACGTTCCGGCTGGCTTTGAGGTTACTCGTGTGCGTCCTTGTGTAGCACCGAAGTAATACGACTCACTATTAGGGAAGACTCCCTCTGAGAAACCAAACGAAACCTAAAGGAGATTAACATTATGGCTAAGAAGATTTTCACCTCTgcgc     <  1:200282/301‑1 (MQ=255)`  
                                                                                                                                                                                                                                                                                                            `gTCAATGACTATGTCAAACGTAAATACAGGTTCACTTAGTGTGGACAATAAGAAGTTTTGGGCTACCGTAGAGTCCTCGGAGCATTCCTTCGAGGTTCCAATCTACGCTGAGACCCTAGACGAAGCTCTGGAGTTAGCCGAATGGCAATACGTTCCGGCTGGCTTTGAGGTTACTCGTGTGCGTCCTTGTGTAGCACCGAAGTAATACGACTCACTATTAGGGAAGACTCCCTCTGAGAAACCAAACGAAACCTAAAGGAGATTAACATTATGGCTAAGAAGATTTTCACctcctgtctct  >  1:217393/1‑293 (MQ=255)`  

                                                                                                                                                                                                                                                                                                            `|`                                                                                                                                                                                                                                                                                                              
`TCGTTCAATGACCGTTGAGCAGTTCAAGGGATACTGCTTCGGTAACATCTTAAAGTACAGACTACGTGCTGGTAAGAAGTCAGAGTTAGCGTACTTAGAGAAAGACCTAGCGAAAGCAGACTTCTATAAAGAACTCTTTGAGAAACATAAGGATAAATGTTATGCATAACTTCAAGTCAACCCCACCTGCCGACAGCCTATCTGATGACTTCACATCTTGCTCAGAGTGGTGCCGAAAGATGTGGGAAGAGACATTCGACGATGCGTACATCAAGCTGTATGAACTTTGGAAATCGAGAGGTCAATGACTATGTCAAACGTAAATACAGGTTCACTTAGTGTGGACAATAAGAAGTTTTGGGCTACCGTAGAGTCCTCGGAGCATTCCTTCGAGGTTCCAATCTACGCTGAGACCCTAGACGAAGCTCTGGAGTTAGCCGAATGGCAATACGTTCCGGCTGGCTTTGAGGTTACTCGTGTGCGTCCTTGTGTAGCACCGAAGTAATACGACTCACTATTAGGGAAGACTCCCTCTGAGAAACCAAACGAAACCTAAAGGAGATTAACATTATGGCTAAGAAGATTTTCACCTCTGCGCTGG  >  NC_001604.1/8588‑9188`

  

Alignment Legend

Aligned base mismatch/match (shaded by quality score): `ATCG/ATCG` `< 3 ≤` `ATCG/ATCG` `< 11 ≤` `ATCG/ATCG` `< 24 ≤` `ATCG/ATCG` `< 36 ≤` `ATCG/ATCG` `< 38 ≤` `ATCG/ATCG`

Unaligned base: `atcg`    Masked matching base: `atcg`    Alignment gap: `‑`    Deleted base: `‑`

Reads not counted as support for junction

read\_name Not counted due to insufficient overlap past the breakpoint.

read\_name Not counted due to not crossing MOB target site duplication.
